# Impact of Obesity on Cardiac Volumes and Left Ventricular Diameter: A Cross-Sectional Study in an Iranian Heart Center

**DOI:** 10.1155/2024/7038875

**Published:** 2024-06-12

**Authors:** Fatemeh Omidi, Mohammad Javad Nasiri, Soheila Sadeghi

**Affiliations:** ^1^Department of Cardiology, Imam Hossein Hospital, Shahid Beheshti University of Medical Sciences, Tehran, Iran; ^2^School of Medicine, Shahid Beheshti University of Medical Sciences, Tehran, Iran; ^3^Clinical Research Development Center, Imam Hossein Educational Hospital, Shahid Beheshti University of Medical Sciences, Tehran, Iran

## Abstract

**Introduction:**

Obesity, a pressing global health issue worldwide, contributes to risk factors such as hypertension and dyslipidemia, creating an unfavorable cardiovascular environment and increasing the likelihood of adverse cardiac events. His study aims to assess the impact of obesity on various cardiovascular parameters.

**Methods:**

A cross-sectional analysis was conducted at a Heart Center, focusing on adults admitted for suspected heart diseases. The dataset included information on demographics, clinical history, laboratory results, and echocardiography. Descriptive analysis and multiple linear regression were employed using IBM SPSS Statistics version 26.

**Results:**

The study of 105 individuals with suspected heart diseases revealed prevalent health factors such as hypertension (47.6%) and hyperlipidemia (61%). Body mass index (BMI) averaged 30, indicating a trend toward overweight. Obesity significantly associated with higher systolic blood pressure (SBP, *p*=0.005) and diastolic blood pressure (DBP, *p*=0.002), larger cardiac volumes (end-diastolic volume, EDV, *p*=0.013; end-systolic volume, ESV, *p*=0.040), and a marginally significant influence on left ventricular end-diastolic diameter (LVEDD, *p*=0.068). No significant associations were found with left ventricular end-systolic diameter (LVEDS), heart rate (HR), or ejection fraction (EF).

**Conclusions:**

Our study highlights a significant association between obesity and elevated blood pressure, larger cardiac volumes, and a marginal impact on left ventricular end-diastolic diameter. While caution is needed in inferring causation due to the study's cross-sectional nature, these findings underscore the importance of addressing obesity as a potential risk factor for adverse cardiovascular outcomes. Further investigations are warranted to enhance our understanding of the complex interplay between obesity and cardiovascular health.

## 1. Introduction

Obesity stands as a pervasive global health challenge, exerting a substantial burden on both public health resources and individuals worldwide [1, 2]. According to the World Health Organization (WHO), more than 1.9 billion adults aged 18 years and older were overweight in 2016, and of these, over 650 million were obese [3]. Its impact spans diverse populations, affecting people across various demographics and socioeconomic backgrounds [4, 5]. Excess body weight contributes to the development of risk factors such as hypertension, dyslipidemia, and insulin resistance [6–9]. These factors collectively create an unfavorable cardiovascular environment, promoting atherosclerosis, coronary artery disease, and ultimately increasing the likelihood of adverse cardiac events [10–13]. Additionally, obesity induces chronic inflammation and oxidative stress, further exacerbating the pathological processes underlying CVDs [14–16].

Existing research has highlighted the adverse cardiovascular outcomes associated with obesity [17–20]. However, the specific impacts of obesity on cardiac function and the nuanced interplay with other factors remain subjects that need exploration. Understanding how obesity independently influences cardiac health is vital for developing targeted interventions and refining risk assessments.

This study aims to uncover potential associations, elucidate the nuanced relationship between obesity and cardiovascular health, and provide insights that can inform personalized interventions and risk assessments.

## 2. Methods

### 2.1. Study Design and Participants

This one-year cross-sectional study, conducted at a University Heart Center in Tehran, Iran, aimed to investigate the intricate relationship between obesity and cardiovascular health in adults admitted with suspected heart diseases. We applied a well-recognized formula tailored to our experimental design, specifically estimating a ratio that considered factors such as effect size, variability, and the desired level of confidence.

The inclusion criteria encompassed adults aged 18 years and above, presenting with suspected heart diseases, and falling within the obesity range based on body mass index (BMI).

The study employed a purposive sampling method and a nonrandomized technique used to select participants based on predefined criteria that align with the study's objectives. The purposive sampling method allowed for the selection of participants who met specific criteria essential for examining the research question, enhancing the study's internal validity, and minimizing bias. This approach ensured that the study population was representative of the target population.

To maintain specificity and minimize confounding factors, exclusion criteria were applied, excluding participants with a history of cardiac procedures, liver or thyroid complications, diabetes, hypertension unrelated to suspected heart diseases, inadequate echocardiographic views, and those with valve stenosis or moderate/severe valvular insufficiency. These exclusion criteria help the study stay focused and controlled, making it easier to understand the specific heart conditions being studied and reducing the chances of confusing factors. Ethical approval was diligently obtained from the Ethics Committee of the Shahid Beheshti University of Medical Sciences, Tehran, Iran, ensuring the study's adherence to ethical standards in human research (IR.SBMU.RETECH.REC.1402.659). We protected participant privacy by removing personal details and securely storing data. Everyone involved consented after understanding the study's purpose, methods, and possible outcomes. Participation was voluntary, and consent forms were easy to understand, encouraging questions for full comprehension.

### 2.2. Data Collection

Comprehensive demographic, physiological, and clinical data were systematically collected from each participant during their initial visit, with a particular focus on variables related to obesity. This encompassed details such as age, gender, height, weight, body mass index (BMI), blood pressure, heart rate, lipid profiles, electrocardiogram (ECG) results, current medical history, and classic risk factors for cardiovascular diseases associated with obesity (including dyslipidemia, smoking, and triad). Medication information, covering lipid-lowering drugs and cardiovascular medications, was also recorded. Subsequently, participants underwent transthoracic echocardiography to measure cardiac components and assess heart function and structure. Trained research personnel followed standardized protocols throughout the process. The collection of data using standardized procedures ensured the accuracy and reliability of the dataset, thereby enhancing transparency and reproducibility in subsequent analyses.

### 2.3. Statistical Analysis

Descriptive analysis and multiple linear regression were employed to examine the relationships between obesity and cardiovascular health measures. Multiple linear regression allowed us to assess the impact of obesity on various cardiovascular measures by examining the relationships between them. Specifically, it helped us understand how changes in obesity levels might predict changes in cardiovascular health outcomes. Descriptive statistics provided an overview of the key parameters, while multiple linear regression assessed the impact of obesity on various cardiovascular measures. Normality tests, such as the Shapiro–Wilk test or Kolmogorov–Smirnov test, were conducted to assess whether the data followed a normal distribution. The statistical analyses were conducted using IBM SPSS Statistics for Windows, version 26 (IBM Corp).

## 3. Results

### 3.1. Baseline Characteristics of Patients

In this cross-sectional study, an analysis was conducted on 105 individuals with suspected heart diseases, examining a range of demographic and clinical parameters within a diverse cohort. The participants, aged 40 to 78 years with a mean age of 58.3 years, displayed a balanced gender distribution, with 51.4% male and 48.6% female. The study provides insights into the prevalence of various health-related factors among the participants. Notably, 47.6% reported hypertension (HTN), while 52.4% did not. Hyperlipidemia (HLP) was observed in 61% of the cohort, contrasting with 39% who reported normal lipid levels. Family history (FH) of cardiovascular diseases showed 17.1% with a positive history and 81.9% without. Coronary artery disease (CS) affected 19% of the population, with 80% exhibiting no signs of this condition. Biochemical analyses revealed a mean total cholesterol (Chol) level of 159.19 mg/dL, high-density lipoprotein (HDL) levels at 45.47 mg/dL, low-density lipoprotein (LDL) levels at 99.59 mg/dL, and hemoglobin (HB) levels at 14.22 g/dL.

### 3.2. Descriptive Statistics for Obesity and Cardiovascular Metrics

Table 1 presents key descriptive statistics for BMI and cardiovascular health measures within the study population. Regarding obesity, 33.3% of the 105 participants reported not having it, while a substantial majority of 66.7% acknowledged its presence. The cohort displayed an average body mass index (BMI) of approximately 30, indicating a trend toward overweight. Systolic and diastolic blood pressures were 105.7 ± 12.1 mmHg and 94.5 ± 8.6 mmHg, respectively. Left ventricular measurements (LVEDD and LVEDS), heart rate (HR), and cardiac volumes (EDV and ESV) are provided, offering crucial insights into the cardiovascular health profile of the cohort. The ejection fraction (EF) averaged 58.7 ± 5.7%, indicative of cardiac pumping efficiency.

### 3.3. Impact of Obesity on Cardiovascular Health

Table 2 displays the results of a multiple linear regression analysis investigating the impact of obesity on various cardiovascular health measures. *β* coefficients indicate the magnitude and direction of the effect of obesity on each cardiovascular parameter. A significant positive association was observed for systolic blood pressure (*β* = 9.28, *p*=0.005) and diastolic blood pressure (*β* = 6.35, *p*=0.002), suggesting that individuals with obesity tend to have higher blood pressure. Left ventricular end-diastolic diameter (LVEDD) showed a marginally significant positive relationship (*β* = 0.14, *p*=0.068), implying a potential influence of obesity on this measure. However, left ventricular end-systolic diameter (LVEDS) and heart rate (HR) did not exhibit significant associations (*β* = 0.11, *p*=0.106; *β* = −1.11, *p*=0.617, respectively). End-diastolic volume (EDV) and end-systolic volume (ESV) displayed significant positive associations (*β* = 9.96, *p*=0.013; *β* = 4.07, *p*=0.040), suggesting that individuals with obesity may have larger cardiac volumes. Ejection fraction (EF) did not show a significant association with obesity (*β* = −0.69, *p*=0.950), indicating that cardiac pumping efficiency may not be significantly affected by obesity in this cohort.

## 4. Discussion

### 4.1. Principle Finding

The key findings revealed a significant association between obesity and elevated systolic and diastolic blood pressure, as well as larger cardiac volumes. Additionally, a marginally significant influence on left ventricular end-diastolic diameter was observed. No significant associations were identified with left ventricular end-systolic diameter, heart rate, or ejection fraction.

### 4.2. Clinical Implications

In addition to the highlighted cardiovascular implications, these findings have broader clinical significance. The association between obesity and elevated blood pressure emphasizes the importance of weight management strategies as a means to potentially mitigate hypertension and its cardiovascular consequences [21–23]. The observed larger cardiac volumes in individuals with obesity suggest potential structural adaptations in response to increased body mass, necessitating further investigation into the functional implications and long-term consequences [24–26]. These findings advocate for a holistic approach to cardiovascular care that includes not only blood pressure management but also comprehensive assessments of cardiac structure and function in individuals with obesity. Clinicians should consider tailored interventions that address both weight management and cardiovascular health to optimize patient outcomes and reduce the overall burden of obesity-related cardiovascular issues.

### 4.3. Limitations

While this study provides valuable insights into the association between obesity and cardiovascular health, certain limitations should be acknowledged. Firstly, the cross-sectional design limits our ability to establish causation or determine the temporal sequence of observed associations. Longitudinal studies would provide a more robust understanding of how obesity influences cardiovascular parameters over time. Secondly, the study focused on a specific cohort admitted with suspected heart diseases, potentially limiting generalizability to broader populations. Additionally, reliance on self-reported data and the absence of dietary or physical activity information may introduce bias and confounding variables. Finally, the study did not explore potential interactions with genetic or environmental factors, which could further elucidate the complexity of the relationship between obesity and cardiovascular health. Future research addressing these limitations would enhance the comprehensiveness and applicability of the findings.

## 5. Conclusions

In summary, our study highlights a significant association between obesity and elevated blood pressure, larger cardiac volumes, and a marginal impact on left ventricular end-diastolic diameter. While caution is needed in inferring causation due to the study's cross-sectional nature, these findings underscore the importance of addressing obesity as a potential risk factor for adverse cardiovascular outcomes. Further longitudinal investigations are warranted to enhance our understanding of the complex interplay between obesity and cardiovascular health. These insights contribute valuable information for developing preventive and therapeutic strategies to mitigate cardiovascular risks associated with obesity.

## Figures and Tables

**Table 1:** Descriptive statistics of BMI and cardiovascular health measures.

Parameters	Mean ± SD	Median (IQR)	Range
BMI	34.9 ± 10.8	34.4 (28.6–41.5)	15.85–76.5
SBP	105.7 ± 12.1	105 (96–113)	78–154
DBP	94.5 ± 8.6	94 (89–101)	82–126
LVEDD	4.8 ± 0.3	4.88 (4.66–5.11)	4.32–5.7
LVEDS	3.1 ± 0.3	3.15 (2.9–3.4)	2.11–3.8
HR	69.4 ± 9.6	69.0 (62.0–75.0)	46.0–106.0
EDV	69.6 ± 17.9	69.5 (57.6–79.6)	31.9–123.5
ESV	27.1 ± 8.8	26.5 (20.8–32.2)	10.6–61.4
EF	58.7 ± 5.7	59.2 (55.9–61.4)	50.3–72.3

BMI: body mass index, SBP: systolic blood pressure (mmHg), DBP: diastolic blood pressure (mmHg), LVEDD: left ventricular end-diastolic diameter, LVEDS: left ventricular end-systolic diameter, HR: heart rate, EDV: end-diastolic volume, ESV: end-systolic volume, EF: ejection fraction.

**Table 2:** Multiple linear regression assessing the impact of obesity on cardiovascular health.

Cardiovascular measures	*β* coefficient	*p* value
SBP	9.28	0.005
DBP	6.35	0.002
LVEDD	0.14	0.068
LVEDS	0.11	0.106
HR	−1.11	0.617
EDV	9.96	0.013
ESV	4.07	0.040
EF	−0.69	0.950

SBP: systolic blood pressure (mmHg), DBP: diastolic blood pressure (mmHg), LVEDD: left ventricular end-diastolic diameter, LVEDS: left ventricular end-systolic diameter, HR: heart rate, EDV: end-diastolic volume, ESV: end-systolic volume, EF: ejection fraction.

## Data Availability

The data used to support the findings of this study are included within the article.
